# Cascade Brillouin Lasing in a Tellurite-Glass Microsphere Resonator with Whispering Gallery Modes

**DOI:** 10.3390/s22082866

**Published:** 2022-04-08

**Authors:** Elena A. Anashkina, Maria P. Marisova, Vitaly V. Dorofeev, Alexey V. Andrianov

**Affiliations:** 1 Institute of Applied Physics of the Russian Academy of Sciences, 46 Ulyanov Street, 603950 Nizhny Novgorod, Russia; marisova.mariya@rambler.ru (M.P.M.); dorofeev@ihps-nnov.ru (V.V.D.); andrian@ipfran.ru (A.V.A.); 2 G.G. Devyatykh Institute of Chemistry of High-Purity Substances of the Russian Academy of Sciences, 49 Tropinin Street, 603950 Nizhny Novgorod, Russia

**Keywords:** microresonator with whispering gallery modes, Brillouin lasing, tellurite-glass microsphere, Q-factor

## Abstract

Brillouin microlasers based on microresonators with whispering gallery modes (WGMs) are in high demand for different applications including sensing and biosensing. We fabricated a microsphere resonator with WGMs from a synthesized high-quality tellurite glass with record high Q-factors for tellurite microresonators (Q ≥ 2.5 × 10^7^), a high Brillouin gain coefficient (compared to standard materials, e.g., silica glasses), and a Brillouin frequency shift of 9 ± 0.5 GHz. The high density of excited resonance modes and high loaded Q-factors allowed us to achieve experimentally cascade Stokes-Brillouin lasing up to the 4th order inclusive. The experimental results are supported by the results of the theoretical analysis. We also theoretically obtained the dependences of the output Brillouin powers on the pump power and found the pump-power thresholds for the first five Brillouin orders at different values of pump frequency detuning and Q-factors, and showed a significant effect of these parameters on the processes under consideration.

## 1. Introduction

One of the in-demand modern trends in detecting various physical quantities, substances, individual molecules, and nano-objects is the use of microresonators with whispering gallery modes (WGMs) [1,2,3,4,5,6,7]. Such microresonators with high Q-factors and small mode volumes are a promising platform for a wide class of optical microdevices [8,9,10,11,12,13]. Microresonators can simultaneously act as an active (or nonlinear) element and a cavity for laser generation, Raman generation, and Brillouin generation [1,14,15,16]; there are numerous sensing and biosensing applications utilizing this peculiarity [1,2]. The stimulated Brillouin scattering (SBS) of light on acoustic waves in microresonators make possible the ultrasensitive detection of gas [17], multiphysical sensing of light, sound and microwaves [18], sensing nano-objects [19] and so on [20]. A micro-optical gyroscope (rotation sensor) utilizing counter-propagating Brillouin waves can be used for high-sensitivity inertial navigation systems [21,22]. Moreover, quantum control of light and sound can be implemented using the SBS effect in microresonators [23]. Note that the SBS effect is successfully implemented in optical fibers for sensing and other important applications [24,25,26,27,28]. In the case of fibers, enormous lengths are required, but the Brillouin powers are also much higher than in the case of microresonators. So, SBS-based sensors in microresonators and fibers are two various branches of Brillouin photonics.

Another well-known trend in modern optics is the search for new materials with special properties for microresonators [29] and optical fibers [30], which is also relevant for Brillouin photonics [20,31]. In the case of glass microsphere resonators, these can be tellurite glasses, which are characterized by higher third-order nonlinearities compared to traditional silica glasses. SBS is a third-order nonlinear optical process [32], and the Brillouin gain coefficient for tellurite glasses is an order of magnitude larger than that for silica glasses [33]. Tellurite glasses are successfully used in nonlinear and laser fiber optics, which makes it possible to achieve conversion of optical radiation in a wide range of parameters based on various effects, including SBS [33,34,35,36,37,38]. As for tellurite-glass microresonators, to date, many research groups have demonstrated lasing in tellurite microspheres doped with different rare-earth ions (see the review [38] and references therein). However, the huge potential of light generation and transformation due to nonlinear optical processes is practically unexplored. The demonstration of the first-order SBS in a tellurite microsphere is known from the literature [39], but the effects due to Kerr and Raman nonlinearities have not been reported, to the best of our knowledge. One of the main reasons may be the relatively low Q-factors of experimental samples. Therefore, an increase in Q-factors, both by improving the quality of tellurite-glass and by improving the manufacturing technology in the presented work led to the 4th-order SBS, and potentially can lead to the observation of Kerr and Raman nonlinear processes.

The purposes of this work are to experimentally demonstrate cascade Brillouin generation in a specially made high-quality tellurite-glass microsphere and theoretically support the experimental results, to explain the main dependences of the processes under consideration, and to predict the SBS features in tellurite microspheres.

## 2. Materials and Methods

### 2.1. Fabrication and Characterization of Tellurite Glass

**Figure 1:**
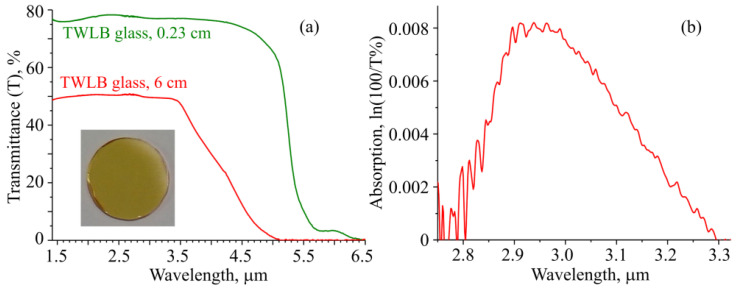
(**a**) Transmittance spectra of produced tellurite-glass samples with photo of 0.23 cm sample in the inset. (**b**) Absorption spectrum within OH band of 6 cm sample.

A microsphere resonator was fabricated from a high-quality tellurite glass with the 64.5TeO_2_–21.5WO_3_–10La_2_O_3_–4Bi_2_O_3_ (TWLB) composition. Tungsten-tellurite glasses modified by lanthanum oxide and bismuth oxide demonstrate excellent optical, physical and chemical properties which make them suitable for fiber-based optical devices [40,41]. The choice of additives to a binary tungsten-tellurite glass was made for the following reasons: lanthanum oxide increases the glass transition temperature and the crystallization resistance of tellurite glasses, and bismuth oxide increases the values of linear (*n*) and nonlinear refractive indices. The TWLB glass was prepared by melting a mixture of initial oxides TeO_2_, WO_3_, La_2_O_3_, and Bi_2_O_3_ in a platinum crucible inside a sealed quartz-glass reactor. The glass synthesis was carried out in an atmosphere of purified oxygen. The low concentration of metal impurities was attained by using high-purity initial oxides and selecting synthesis conditions that minimized contamination of the glass-forming melt with reactor materials [42]. Samples of glasses were made from super-high-purity tellurium oxide obtained by vacuum distillation according to the original method using the high-purity, commercially available reagents WO_3_, La_2_O_3_, and Bi_2_O_3_. The use of high-purity oxides allowed us to obtain glasses with a total content of 3d transition metals of the order of 1–2 ppm wt. To reduce the content of hydroxyl groups in the glass, the samples were synthesized and molded in a sealed quartz-glass reactor equipped with manipulators for mixing the melt and molding samples (for details please see [40,41,42]). A systematic approach to preventing the penetration of impurities guarantees a high purity of synthesized samples [43]. After cooling and annealing at the glass transition temperature, the casting was cut, and the segments were ground and polished for research. The prepared samples were visually optically homogeneous and free from defects (Figure 1a, inset). 

The measured refractive index of the produced TWLB glass was *n* = 2.054 at 1.539 µm.

Next, we measured the transmittance spectra of tellurite-glass samples with a length of 6 cm and a thickness of 0.23 cm in the IR spectral range (Figure 1a). The transmittance of about 78% in the 1.5–4.5 µm range for the 0.23 cm sample was explained by the Fresnel losses at the ends, and the lower transmittance of about 50% in the 1.5–3.5 µm range for long sample was additionally explained by the large divergence of the light beam in our spectrometer and its output through the side surface of the 6 cm TWLB sample. The spectra of the tellurite glasses obtained under ordinary conditions, in air or in undried oxygen, always show pronounced main hydroxyl bands at about 3 and 4.4 µm, as well as combination bands near 1.5 and 2.3 µm. In our case, when the glass-forming melt was dehydrated in dry oxygen, there were no absorption bands of hydroxyl groups in the spectrum of the thin sample. In the spectra of the sample with a length of 6 cm, weakly noticeable hydroxyl main bands were detected; the combination absorption bands with maxima at ~1.5 and 2.3 µm were indistinguishable.

The sample length *L* = 6 cm allowed us to process the 3 µm absorption band and calculate the OH volume-absorption coefficient and OH concentration. The method of calculation is described in detail in [41]. The absorption spectrum in the 3 µm hydroxyl band (corrected with allowance for the baseline) is shown in Figure 1b. The maximum of the band corresponds to a very low absorption value of 0.008 of the whole sample. Features of glass manufacturing which allow the yield of an extremely low content of hydroxyl groups are discussed in the articles [40,41,42]. To estimate the volume-absorption coefficient *α*, we took into account the absorption by hydroxyl groups at both sample ends (*β*~0.0035–0.004 for tungsten-tellurite glasses [40,41]). The volume-absorption coefficient *α* was estimated from the expression ln(*I*_0_/*I*) = *β* + *αL*. We obtained *α* < 0.001 cm^−1^, which corresponds to the extremely low OH concentration of about 5 × 10^15^ cm^−3^ [41]. We drew a single-index fiber with a diameter of 100 µm from the synthesized TWLB glass without any coating. The low content of impurities and OH groups is very important for the fabrication of microresonators with high Q-factors.

### 2.2. Fabrication of Tellurite Microsphere Resonators

In our earlier studies we fabricated tellurite microspheres from single-index fibers using a microheater [44,45]. In this work, we used a CO_2_ laser (Coherent Diamond C-40A), which allowed us to improve the quality of the samples and achieve record loaded Q-factors for tellurite microspheres (≥2.5 × 10^7^). As far as we know, the highest previously measured value of the loaded Q-factor in a tellurite microsphere was 1.07 × 10^7^ and corresponded to an intrinsic recalculated Q-factor of 1.3 × 10^7^ [39].

**Figure 2:**
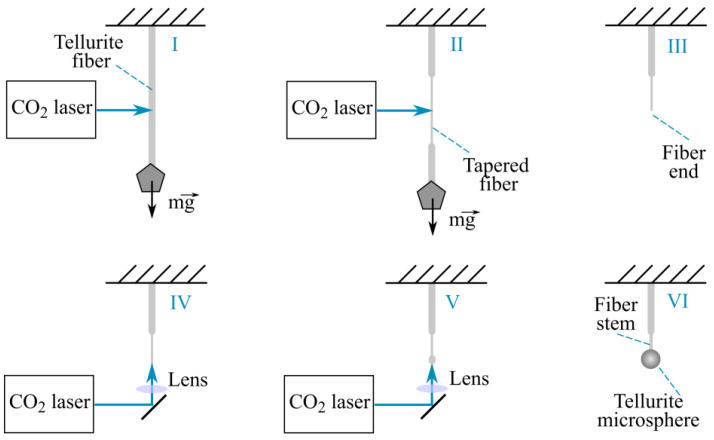
Schematic representation of the successive stages of fabrication of microresonators from single-index tellurite fiber.

The successive stages I–VI of microsphere fabrication are shown in Figure 2. A tellurite single-index fiber with a small weight at the end was vertically suspended (stage I). Then, CO_2_ laser radiation with adjustable pulse duration (0.5–1 s) and instantaneous power of 2 W was forwarded to the fiber, as a result of which a taper was formed (II) and then broke (III). After that, the CO_2_ laser pulsed with a 100 ms duration and 50–100 mJ energy were focused on the formed fiber end (IV), which was heated and melted with the formation of a sphere under the action of the surface-tension force (V). The final size of the microsphere at the end of the fiber stem was controlled by changing the laser parameters and could range from ~30 µm to a few hundred µm. In this study, we used a microsphere with a diameter of 75 μm. 

### 2.3. Theoretical Description of Microsphere Characteristics

We theoretically calculated the characteristics of the fabricated tellurite microsphere, which are important for the analysis of cascade Brillouin lasing. The eigenfrequencies of the spherical microresonator were found from the characteristic equations obtained from the system of Maxwell’s equations with allowance for the boundary conditions [46]:

(1) $$\frac{{\left[{\left(nk_{0}R\right)}^{1/2}J_{l+1/2}\left(nk_{0}R\right)\right]}^{\prime }}{{\left(nk_{0}R\right)}^{1/2}J_{l+1/2}\left(nk_{0}R\right)}=n^{p}\frac{{\left[{\left(k_{0}R\right)}^{1/2}H_{l+1/2}^{\left(1\right)}\left(k_{0}R\right)\right]}^{\prime }}{{\left(k_{0}R\right)}^{1/2}H_{l+1/2}^{\left(1\right)}\left(k_{0}R\right)},$$

where *p* = 1 for the transverse magnetic (TM) WGMs, *p* = −1 for the transverse electric (TE) WGMs, *k*_0_ = 2π*f*/*c*, *R* is the microsphere radius, *n* is the glass refractive index, *f* is the frequency, *c* is the vacuum speed of light, *l* is the polar mode index, *J*_ν_ is the Bessel function of order ν, *H*_ν_^(1)^ is the Hankel function of the 1st order ν, and the prime denotes the derivative with respect to the argument in the parentheses. Each equation has multiple roots *f_q_*, and when sorted in ascending order, *q* ≥ 1 corresponds to the radial mode index. The characteristic equations were solved numerically using a home-made computer python code; the glass dispersion *n*(*f*) was taken into account. The roots were iteratively localized using high-order approximations [47]. For the fundamental WGM (*q* = 1, *l* = *m*), the free spectral range (FSR) was FSR = *f_l_*_+1_ − *f_l_* ≈ 600 GHz near *λ* = 1.55 μm.

**Figure 3:**
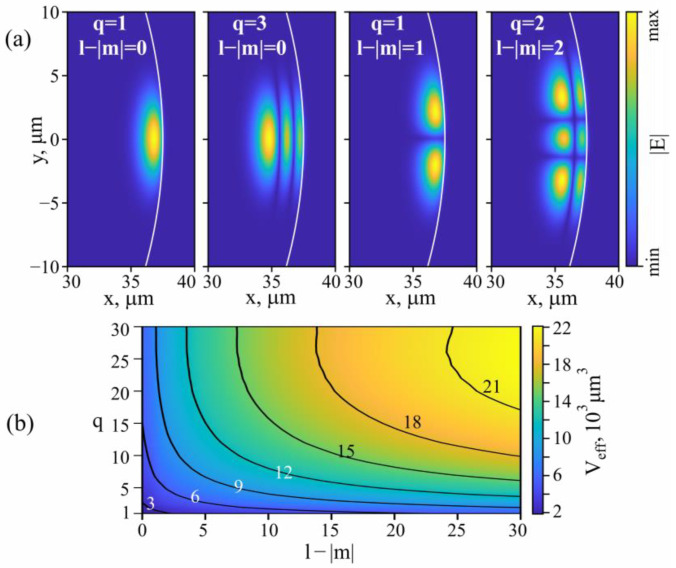
(**a**) Examples of calculated electric fields of eigenmodes with different indices for 75 µm tellurite microsphere resonator. (**b**) Calculated effective mode volume for eigenmodes with different indices for this resonator.

The expressions for the eigenmode fields are cumbersome and are not presented here but can be found, for instance, in [48]. The examples of the calculated fields for eigenmodes with different indices (for better understanding of the meaning of the indices *q* and *l* − |*m*|) are shown in Figure 3a.

We used the expressions for fields from [48] for calculating the effective mode volumes *V*_*eff*_ (below they will be used for estimating intracavity Brillouin gain coefficients):

(2) $$V_{eff}=2\pi R\;\frac{{\left(\int S_{\varphi }d^{2}r\;\right)}^{2}}{\int S_{\varphi }^{2}d^{2}r\;}.$$

In an ideal microsphere for a given *l*, modes with different azimuthal indices *m*, −*l* ≤ *m* ≤ *l* are degenerate. This degeneracy is lifted if the microresonator is deformed. The resulting mode splitting can be described by the perturbation theory and, in the simplest case of a deformation into a spheroid, new eigenfrequencies can be found as follows [49]:

(3) $$\frac{f_{l,m}}{f_{l}^{\left(0\right)}}=1-\frac{1}{3}\eta \left(1-3\frac{m^{2}}{l\left(l+1\right)}\right)=1,$$

where *f*_*l*,*m*_ is the split eigenfrequency, *f_l_*^(0)^ is the unperturbed eigenfrequency from Equation (1), $\eta =\left(R_{z}-R_{x}\right)/R$ is the shape-deformation parameter, *R_z_* and *R_x_* are the spheroid semiaxes (*z* is the symmetry axis). 

### 2.4. Theoretical Model of Cascade Brillouin Lasing

**Figure 4:**
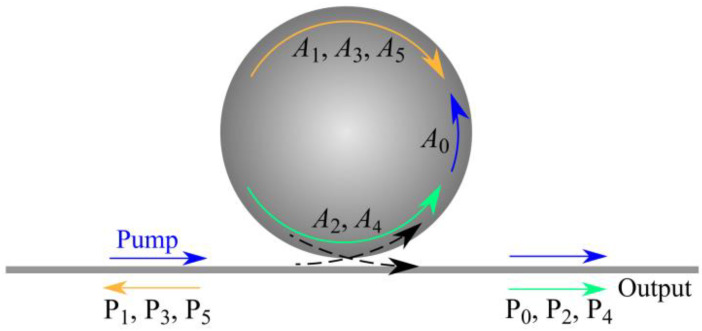
Schematic diagram of cascade Brillouin lasing in microsphere. Even-order Brillouin waves propagate in forward direction and odd-order Brillouin waves propagate in backward direction relative to pump wave. *A*j is intracavity field amplitude, *P*j is output power, subscript j indicates the jth order of the Brillouin cascade, j = 0 corresponds to pump wave. Only Brillouin orders considered in this study are indicated.

To support the experimental results on cascade Brillouin lasing in the fabricated tellurite microsphere resonator (presented below in Section 3.1), to get a deeper insight into the features of this process and predict important characteristics, such as Brillouin laser thresholds (*P*^th^), dependence of output powers on pump power, and the number of Brillouin cascades, we performed the theoretical analysis. The schematic diagram of the considered cascade Brillouin laser is shown in Figure 4, where the pump laser initiates the generation of a backward Stokes–Brillouin wave that acts as a pump for the Stokes–Brillouin wave of the second cascade. The 2nd-order Brillouin wave propagates in the opposite direction with respect to the 1st-order wave. Further, the 2nd-order Brillouin wave pumps the 3rd-order Brillouin wave, and so on. In this case, even-order Brillouin waves propagate in the forward direction, while odd-order Brillouin waves propagate in the backward direction relative to the pump.

The theoretical study of cascade Brillouin lasing according to the scheme shown in Figure 4 was performed using the mean-field model and the coupled mode theory [50,51]:

(4) $$\frac{dA_{0}}{dt}=\left(i\Delta \omega_{0}-\frac{1}{2\tau_{0}}\right)A_{0}-g_{1}{\left|A_{1}\right|}^{2}A_{0}+\sqrt{\kappa_{0}}S,$$

(5) $$\frac{dA_{j}}{dt}=-\frac{1}{2\tau_{j}}A_{j}+g_{j}{\left|A_{j-1}\right|}^{2}A_{j}-g_{j+1}{\left|A_{j+1}\right|}^{2}A_{j},\;\;\;j=1,\ldots N-1,$$

(6) $$\frac{dA_{N}}{dt}=-\frac{1}{2\tau_{N}}A_{N}+g_{N}{\left|A_{N-1}\right|}^{2}A_{N},$$

where *A*_0_ is the intracavity field amplitude at the pump frequency; *A*_j_ is the intracavity field amplitude of the generated Brillouin wave of the jth order (j = 1,…N); N is the maximum order of the generated Brillouin cascade; *t* is time; Δω_0_ is the detuning of the pump frequency from the exact resonance; *S* is the amplitude of the incident pump wave, *P_p_* = |*S*|^2^ is the pump power; *τ*_j_ is the effective photon lifetime (related to the loaded Q-factor by *Q*_j_ = *ω*⸱*τ*_j_) with allowance for the intrinsic lifetime *τ*_j_^0^ and coupling lifetime *τ*_j_*^c^* (1/*τ*_j_ = 1/*τ*_j_^0^ + 1/*τ*_j_^c^) for the WGM in which the jth-order Brillouin lasing arises (hereinafter the subscript j = 0 corresponds to the pumped WGM); *κ*_j_ = 1/*τ*_j_*^c^* is the coupling coefficient; $g_{j}=\Gamma_{j}g_{Te}c^{2}/\left(2n^{2}V_{j}\right)$ is the intracavity Brillouin gain coefficient; $g_{Te}$ is the Brillouin gain for bulk tellurite glass ($g_{Te}$ ≈ 1.7⸱10^−10^ m/W [33]); *n* is the refractive index; *V*j is the effective mode volume of the WGM in which the jth-order Brillouin lasing arises; and *Γ*_j_ is the overlap integral between the mode fields corresponding the jth and (j − 1)th Brillouin cascade. The output power is *P*_j_ = *κ*|*A*_j_|^2^. 

For the theoretical analysis we took *τ*_j_^c^ = 4*τ*_j_ based on our experimental estimates. The effective mode volumes were set *V*_j_ = 10^4^ µm^3^ (based on the results presented in Figure 3b) and *Γ*_j_ = 0.05, therefore, $g_{j}$ ≈ 9⸱10^18^ 1/(W⸱s^2^). 

We considered steady-state processes, so d*A*_j_/d*t* = 0 in Equations (4)–(6) and the system of equations describing cascade Brillouin lasing is algebraic. For the successively analyzed N = 1, …, N = 5, the system of Equations (4)–(6) was easily solved analytically and expressions for |*A*_j_|^2^ and threshold pump powers *P*_j_^th^ were found.

## 3. Results

### 3.1. Experimental Results

**Figure 5:**
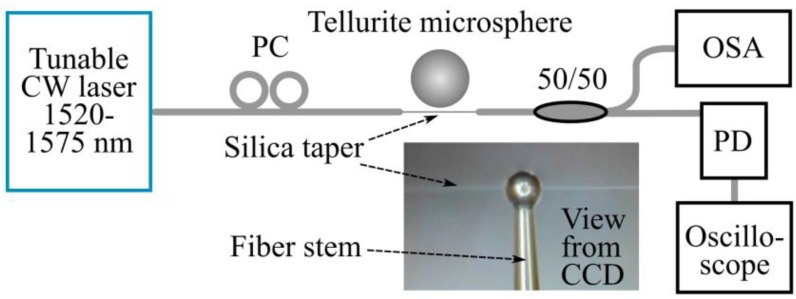
Simplified schematic diagram of the experimental setup. Inset: microphoto of the used tellurite microsphere and silica fiber taper.

The experiments with the considered microsphere were carried out according to the scheme shown in Figure 5. Like in our previous works [44,45,52,53], the study was conducted in an acrylic glove box to minimize the influence of dust, air currents, and other external factors. The WGM microsphere was excited by a tunable CW narrow-band telecom laser (Pure Photonics, 18 dBm, 10 kHz linewidth, and 190.3–197.9 THz tuning spectral range) controlled by a computer. Laser radiation was coupled into the microsphere resonator through a silica taper with a diameter of ~3 μm. The taper was made by heating and stretching a standard telecom fiber as in [52,53]. Before the taper, a polarizing controller (PC) was used in the circuit. The microsphere was aligned relative to the taper by a computer-controlled micropositioner with a piezoelectric drive. CCD cameras also placed in an acrylic glove box were used for visualization. The same taper was used to extract the converted radiation from the microsphere resonator; therefore, the output signal also contained unconverted pump-laser radiation.

**Figure 6:**
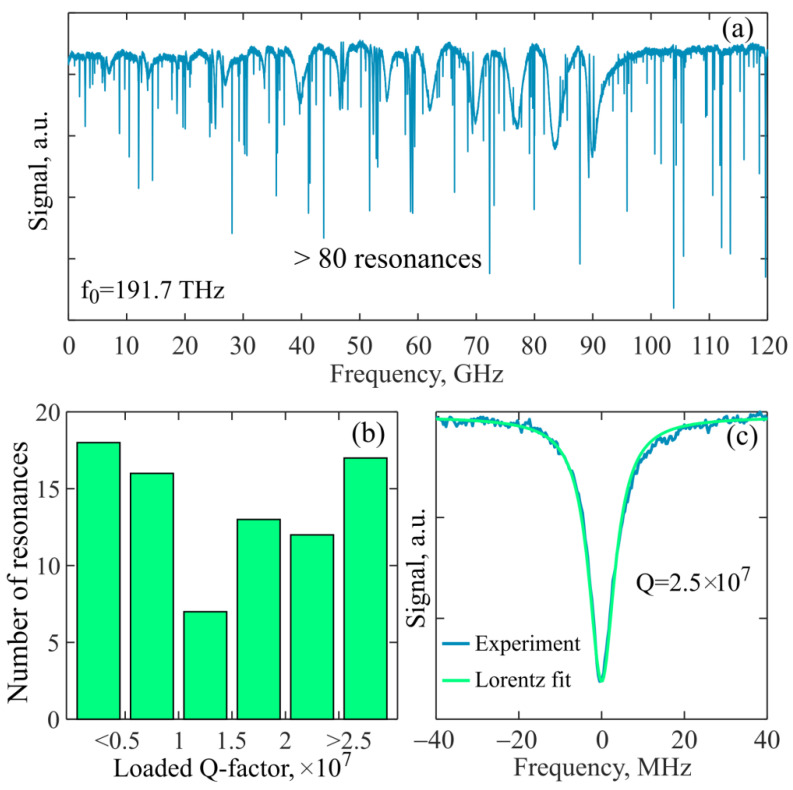
(**a**) Experimental resonance dips of eigenmodes of the produced 75 µm tellurite microsphere resonator recorded for the output pump power of 0.3 µW with the oscilloscope at the pump-laser-sweeping rate of 10 GHz/s. (**b**) Statistics of Q-factors for these resonances. (**c**) Resonance dip on magnified scale and its Lorentz approximation demonstrating loaded Q-factor.

First, we measured the Q-factors of the WGMs for this microresonator. The pump-laser frequency was swept with a rate of 10 GHz/s in the range of 150 GHz, and the transmitted signal was fed through a photodetector to an oscilloscope (Figure 5). The laser power was attenuated to ~0.3 µW to avoid nonlinear optical and thermo-optical effects and to measure linear resonances. The data from the oscilloscope, recalculated to the frequency domain, showed a large number of resonant modes: >80 over the 120 GHz (FSR/5) frequency range (Figure 6a). With the help of specially developed matlab code, we processed this spectrum and determined the loaded Q-factors for each resonance. The statistics are given in Figure 6b. Note that 60% of the resonances had a loaded Q-factor > 1 × 10^7^, and 20% of the resonances had a loaded Q-factor of more than 2.5 × 10^7^. The resonance curves were symmetrical and well approximated by the Lorentz function. An example of a resonance with a Q-factor of 2.5 × 10^7^ is shown in Figure 6c. The accuracy of Q-factor measurements was estimated to be 5% for most resonances, which was limited by resonance-curve fitting uncertainties that originated from the noise and slight deviations of measured shapes from the perfect Lorentz curves.

**Figure 7:**
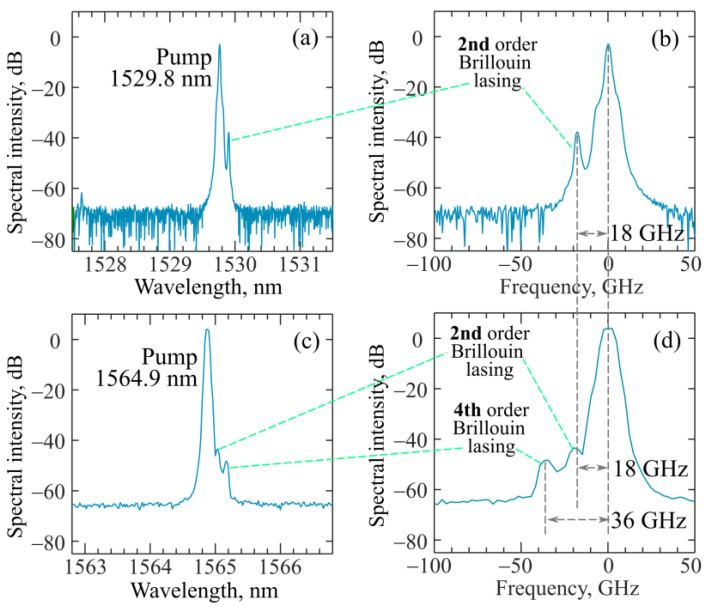
Experimental spectra measured for waves propagating co-directionally with pump. Spectra demonstrating cascade Stokes–Brillouin lasing: (**a**,**b**) of the 2nd order; (**c**,**d**) up to the 4th order.

Next, we experimentally achieved cascade Brillouin generation in various families of WGMs in the fabricated microsphere. We measured the spectra at the output of the taper with an optical spectrum analyzer (OSA). Only even-order Brillouin waves could be recorded in our scheme. The OSA resolution was 0.02 nm and the wavelength accuracy was 0.01 nm (~2 GHz), which made it possible to identify different Brillouin cascades and measure the Brillouin shift, but did not allow accurate measurement of the individual narrow-band spectral line shapes of Brillouin lasing. By tuning the pump-laser wavelength, due to the high WGM density (Figure 6a), we experimentally attained the excitation of suitable modes, for which there were other modes that were spectrally separated by the Brillouin shift Δ*_B_*. For example, at a pump wavelength of 1529.8 nm, we observed 2nd-order Stokes–Brillouin wave generation shifted by 18 ± 2 GHz relative to the pump frequency (Figure 7a,b). The best experimental result was attained at a pump wavelength of 1564.9 nm. For this case, we recorded the spectrum of generated Stokes–Brillouin waves of the 2nd and 4th orders to be shifted, respectively, by 18 ± 2 GHz and 36 ± 2 GHz relative to the pump (Figure 7c,d). Thus, the value of the Brillouin shift corresponded to Δ*_B_* = 9 ± 0.5 GHz. In [39], the Brillouin shift was Δ*_B_* = 8.2 GHz for zinc–tellurite glass. In our case, we used glass of the tungsten-tellurite system with slightly different physical properties, which explains the difference in the Δ*_B_* values. Note that the achievement of cascade Brillouin generation of the 4th order is possible here due to the high WGM density and the high quality of the fabricated microsphere, which provides high values of the loaded Q-factors for most of the resonant modes. The high WGM density makes it possible to select a few modes with a spectral distance of Δ*_B_* required for cascade Brillouin lasing.

### 3.2. Theoretical Results

**Figure 8:**
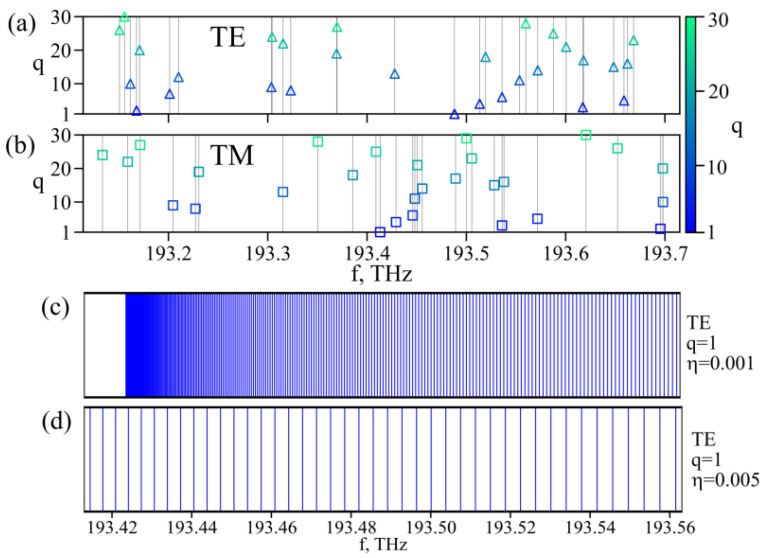
Eigenfrequencies of ideal 75 µm tellurite microsphere near λ = 1.55 μm for TE (**a**) and TM (**b**) modes with different radial indices *q*; vertical lines show resonance positions. Resulting splitting of the fundamental TE mode for microresonator with the shape-deformation parameter *η* defined based on Equation (3); *η* = 0.001 (**c**), *η* = 0.005 (**d**).

First of all, we qualitatively explained the high density of the resonant dips that were experimentally observed in Figure 6a. We calculated the eigenfrequencies of the TE and TM modes for different radial indices *q* in the case of a perfectly symmetrical spherical resonator (see Section 2.3, Equation (1)). Figure 8a,b show the eigenfrequencies in the spectral range corresponding to the FSR. Since modes with different azimuthal indices *m* (−*l* ≤ *m* ≤ *l*) are degenerate for an ideal spherical resonator, the number of modes in Figure 8a,b is relatively small (total 60 for TE and TM modes for the considered *q* = 1…30 in the spectral range corresponding to FSR = 600 GHz). However, due to the slight deformation of the resonator and the deviation from ideal symmetry, degeneracy is lifted. We estimated the shift of the eigenfrequencies from Equation (3) for the radial mode with *q* = 1 for a spheroid for two different shape-perturbation parameters. The removal of the degeneracy of eigenfrequencies is shown in Figure 8c,d in the spectral range of about FSR/4. It is seen that the mode density increases significantly. The stronger the shape perturbation, the larger the frequency shift. Frequency splitting occurs in a similar way for modes with other *q* indices. Therefore, a large number of WGMs can indeed be excited in the system. The excitation efficiency depends on the overlap integrals of the evanescent field of a fiber taper with the field of a certain mode. WGMs with large *q* and *l* − |*m*| indices are practically not excited. Thus, the calculation results presented in Figure 8a–d qualitatively explain the experimental results observed in Figure 6a (although in the experiment we could not distinguish between the TE and TM modes)

**Figure 9:**
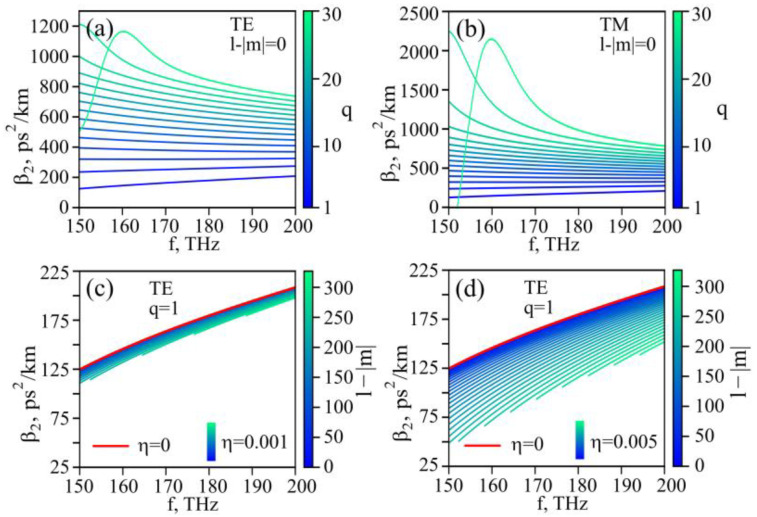
(**a**,**b**) 2nd-order dispersion of TE (**a**) and TM modes (**b**) of ideal microsphere as a function of frequency; only modes with odd q are shown. (**c**,**d**) 2nd-order dispersion of TE modes with one radial variation for microresonator with η = 0.001 ((**c**), every 20th mode is shown), η = 0.005 ((**d**), every 10th mode is shown); red line marks the dispersion of the corresponding modes of ideal microsphere.

Then, we calculated the 2nd-order dispersion *β*_2_ for different modes. For the steady-state Brillouin lasing investigated here, the dispersion itself is not important. However, the dispersion can strongly influence other nonlinear processes occurring in the microsphere, for example, due to the Kerr nonlinearity. Therefore, the calculation of the dispersion is important for the complete characterization of the microsphere properties. We found that the dispersion was normal for all considered modes (Figure 9a–d).

**Figure 10:**
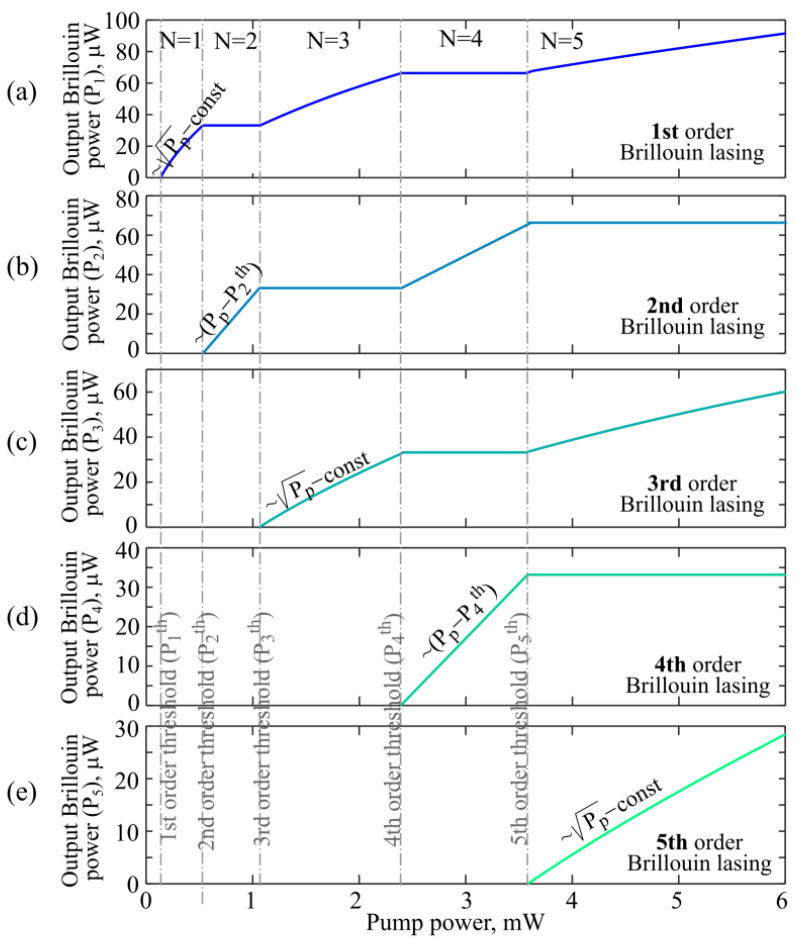
Theoretically calculated output powers of generated Stokes–Brillouin waves of the 1st order (**a**), 2nd order (**b**); 3rd order (**c**); 4th order (**d**); and 5th order (**e**) as functions of pump power for zero detuning (Δω_0_ = 0) and loaded Q-factors *Q* = 2.5 × 10^7^.

Next, we studied steady-state cascade Brillouin lasing in the framework of the system of Equations (4)–(6). We successively considered the cases N = 1, …, N = 5, and for each specific *N* we found the dependences of the output power of the jth cascade on the pump power (j = 0…N). Since in the experiment the maximum observed even Brillouin order was 4 (the 6th cascade was not observed), our theoretical analysis was limited to the maximum value N = 5, but this approach can be applied to any N. The output powers of Stokes–Brillouin waves of the 1st, 2nd, 3rd, 4th, and 5th orders as functions of the pump power are plotted in Figure 10a–e, respectively, for the case of exact resonance (zero detuning Δω_0_ = 0) and loaded Q-factor *Q* = 2.5 × 10^7^. When N is even, the powers of odd Brillouin orders are constant and do not depend on the pump power for *P*_N−1_^st^ < *P_P_* < *P*_N_^th^, while the powers of even Brillouin orders increase linearly with increasing pump power. When N is odd, the powers of even Brillouin orders are constant and do not depend on the pump power for *P*_N−1_^st^ < *P_P_* < *P*_N_^th^, while the powers of odd Brillouin orders increase according to the law $\sim \sqrt{P_{p}}-const$. Analytical dependences presented in Figure 10b,d also demonstrate that the power in the 2nd Brillouin cascade is twice as high as in the 4th cascade. This result agrees with the experimental data presented in Figure 7c,d.

**Figure 11:**
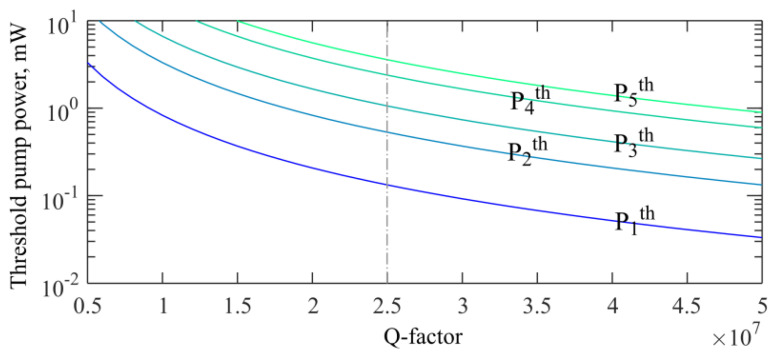
Theoretically calculated threshold pump powers as functions of Q-factors for cascade Brillouin waves of the 1st–5th orders for zero detuning (Δω_0_ = 0). Thresholds for waves of the 6th and higher orders are not shown.

Further, we found that Q-factors strongly affect cascade Brillouin lasing, and the decrease in Q-factors leads to a significant increase in the pump-power thresholds. The threshold pump powers as a function of the Q-factor are plotted in Figure 11 for the 1st–5th cascades, assuming zero detuning (Δω_0_ = 0). 

**Figure 12:**
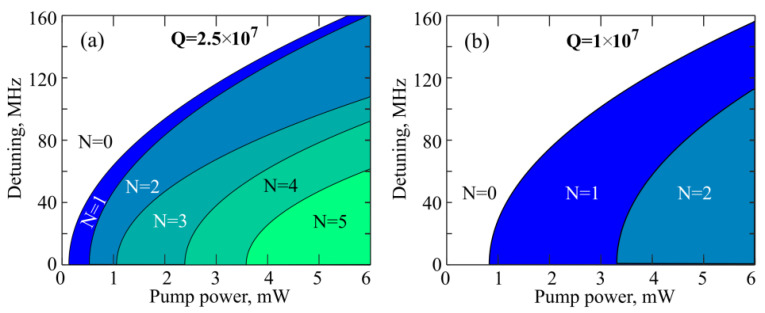
Theoretically calculated diagram demonstrating the number of Brillouin laser cascades for different pump powers and detuning for loaded *Q*-factors: *Q* = 2.5 × 10^7^ (**a**) and *Q* = 1 × 10^7^ (**b**).

Finally, we calculated the diagram demonstrating the number of cascades at different values of the pump power and detuning for two Q-factor values (Figure 12a,b). Note that the exact solution of the system of Equations (4)–(6) gives *P*_2_^nd^ − *P*_1_^st^ = *const* and *P*_4_^th^ − *P*_3_^rd^ = *const* for any detuning. For each Q-factor, the larger the detuning, the higher the threshold for a certain cascade, which agrees with the theoretical results on Brillouin lasing obtained for ring resonators [50]. The Q-factor and detuning significantly affect the number of generated cascades. Indeed, to generate high-order cascade Brillouin waves, a large Q-factor is required. So, the calculations confirmed that the experimentally attained 4th order Brillouin lasing (Figure 7c,d) was possible due to the record high Q-factors of the produced microsphere (for microresonators made of tellurite glasses). 

## 4. Discussion

In this work, we fabricated a 75 µm tellurite microsphere resonator with record high Q-factors for tellurite microresonators. For our sample, 20% of the measured resonances had loaded Q-factors Q ≥ 2.5 × 10^7^ and 60% of resonances had loaded Q-factors Q ≥ 1 × 10^7^. The use of a specially synthesized high-quality ultra-dry glass (64.5TeO_2_–21.5WO_3_–10La_2_O_3_–4Bi_2_O_3_) and the fabrication of microspheres employing a CO_2_ laser instead of a microheater allowed us to increase the Q-factors by an order of magnitude compared to our earlier results [45]. In the produced microresonator, cascade Stokes–Brillouin generation up to the 4th order inclusive was attained. To the best of our knowledge, stimulated Brillouin scattering of the 1st order in a tellurite microsphere was previously reported only in one article [39], where a sample with a loaded Q-factor of 1.07 × 10^7^ was made. Our experimental result, confirmed by the theoretical analysis, was achieved due to the high Q-factors. Cascade Brillouin lasing was observed in different families of WGMs, and a large number of modes with the required spectral interval of 9 GHz corresponding to the Brillouin frequency shift Δ*_B_* was observed experimentally and can be mainly explained by the splitting of degenerate frequencies under a small deformation of the microsphere. As a result of the theoretical analysis, we found the pump-power thresholds for the first five Brillouin orders at different values of detuning *Δω*_0_ and Q-factors and showed a significant influence of these parameters on the processes under consideration. We obtained the dependences of the output Brillouin powers on the pump power. The theoretical analysis of steady-state generation was carried out within the framework of the equations for the mean fields and the theory of coupled modes [50,51]. Note that similar equations are also used to describe laser generation and Raman generation in microresonators and provide a good agreement with the corresponding experimental results [14,44,45,54]. So, the results of our work demonstrate the prospects of using tellurite-glass microspheres for cascade Brillouin lasing, which can expand the scope of microresonator sensors. The obtained results on generating Brillouin cascades can be useful for rotation sensors requiring counter-propagating waves.

## Data Availability

Data underlying the results presented in this article may be obtained from the authors upon reasonable request.
